# A simple method that enhances minority species detection in the microbiota: 16S metagenome-DRIP (Deeper Resolution using an Inhibitory Primer)

**DOI:** 10.20517/mrr.2022.08

**Published:** 2022-05-30

**Authors:** Aruto Nakajima, Keisuke Yoshida, Aina Gotoh, Toshihiko Katoh, Miriam N. Ojima, Mikiyasu Sakanaka, Jin-Zhong Xiao, Toshitaka Odamaki, Takane Katayama

**Affiliations:** ^1^Graduate School of Biostudies, Kyoto University, Sakyo-ku, Kyoto 606-8502, Japan.; ^2^Next Generation Science Institute, Morinaga Milk Industry Co. Ltd., Zama, Kanagawa 252-8583, Japan.

**Keywords:** 16S metagenome-DRIP, α-Type DNA polymerase, biotinylated inhibitory primer, minority species, streptavidin-beads

## Abstract

**Aim:** 16S rRNA gene-based microbiota analyses (16S metagenomes) using next-generation sequencing (NGS) technologies are widely used to examine the microbial community composition in environmental samples. However, the sequencing capacity of NGS is sometimes insufficient to cover the whole microbial community, especially when analyzing soil and fecal microbiotas. This limitation may have hampered the detection of minority species that potentially affect microbiota formation and structure.

**Methods:** We developed a simple method, termed 16S metagenome-DRIP (Deeper Resolution using an Inhibitory Primer), that not only enhances minority species detection but also increases the accuracy of their abundance estimation. The method relies on the inhibition of normal amplicon formation of the 16S rRNA gene of a target major (abundant) species during the first PCR step. The addition of a biotinylated primer that is complementary to the variable sequence of the V3-V4 region of the target species inhibits a normal amplification process to form an aberrant short amplicon. The fragment is then captured by streptavidin beads for removal from the reaction mixture, and the resulting mixture is utilized for the second PCR with barcode-tag primers. Thus, this method only requires two additional experimental procedures to the conventional 16S metagenome analysis. A proof-of-concept experiment was first conducted using a mock sample consisting of the genomes of 14 bacterial species. Then, the method was applied to infant fecal samples using a *Bifidobacterium*-specific inhibitory primer (*n* = 11).

**Results: **As a result, the reads assigned to the family *Bifidobacteriaceae* decreased on average from 16,657 to 1718 per sample without affecting the total read counts (36,073 and 34,778 per sample for the conventional and DRIP methods, respectively). Furthermore, the minority species detection rate increased with neither affecting Bray-Curtis dissimilarity calculated by omitting the target *Bifidobacterium* species (median: 0.049) nor changing the relative abundances of the non-target species. While 115 amplicon sequence variants (ASVs) were unique to the conventional method, 208 ASVs were uniquely detected for the DRIP method. Moreover, the abundance estimation for minority species became more accurate, as revealed thorough comparison with the results of quantitative PCR analysis.

**Conclusion:** The 16S metagenome-DRIP method serves as a useful technique to grasp a deeper and more accurate microbiota composition when combined with conventional 16S metagenome analysis methods.

## INTRODUCTION

Minority species in microbiotas potentially play a crucial role in community structuring. Such species are called keystone species, which are defined as species that occupy a small percentage of an ecosystem but have a disproportionately large impact on the whole community^[1,2]^. Recent studies suggest that bacteria possessing genes (enzymes) with unique metabolic features can serve as keystone species by altering the fitness of other community members^[3-5]^. Thus, to better understand the mechanism of microbial community formation and identify keystone species involved in the process, precise microbiota analyses that detect a wide range of taxa with accurate abundance estimation are necessary. Next-generation sequencing (NGS) technologies have enabled us to obtain several million sequencing reads per run. However, as environmental samples such as soil and feces generally contain trillions of microbes that comprise more than 100 species^[6,7]^, the detection of minority species in such samples remains a challenge. In NGS technologies, most of the sequence reads are necessarily allocated to the majority species; if such reads are reduced, the conserved read space can be used for sequencing other species, thereby increasing the possibility of detecting minority species.

Here, we describe a simple, low-cost method that improves not only the detection of minority species but also the accuracy of their relative abundance estimation in environmental samples. This method, termed 16S metagenome-DRIP (Deeper Resolution using an Inhibitory Primer), only requires two additional experimental procedures to the conventional 16S metagenome analysis. A conceptualization of the method, a proof-of-concept experiment, and an application of the method to infant stool samples are reported.

## METHODS

### Human samples

Fecal samples of 11 infants (6 males and 5 females, average age 5.2 ± 2.7 months, age range between 2 and 11 months), which were collected in our previous study^[8]^, were re-analyzed in this study (see the "*Ethical approval and consent to participate*" section). The fecal DNA was extracted as described previously^[8]^.

### Bacterial culture and genomic DNA extraction

*Bacteroides fragilis* JCM 11019, *Bacteroides ovatus* JCM 5824, *Bacteroides uniformis* JCM 5828, *Bifidobacterium breve* JCM 1192,* Bifidobacterium longum* subspecies *longum *JCM 1217, *Bifidobacterium pseudocatenulatum* JCM 1200, *Blautia wexlerae* JCM 17041, *Collinsella aerofaciens* JCM 10188, *Coprococcus comes* JCM 31264,* Faecalibacterium prausnitzii* JCM 31915, *Lactobacillus gasseri* JCM 1131, *Limosilactobacillus reuteri *JCM 1112, and *Phocaeicola vulgatus* JCM 5826 were obtained from the Japan Collection of Microorganisms (RIKEN BioResource Research Center, Tsukuba, Japan). *Escherichia coli *MG1655 is a laboratory stock. The strains were grown in Gifu Anaerobic Medium (GAM; Nissui Pharmaceutical, Tokyo, Japan) at 37 °C under anaerobic conditions using the AnaeroPack system (Mitsubishi Gas Chemical Co., Tokyo, Japan). The cells were harvested by centrifugation and subjected to genomic DNA extraction as described previously^[9]^.

### Microbiota analysis

#### The first PCR

The V3-V4 region of the 16S rRNA gene was amplified with PrimeSTAR Max DNA polymerase (TaKaRa Bio, Shiga, Japan) (see the "*Results*" Section). Tru357F (5′-CGCTCTTCCGATCTCTGTACGGRAGGCAGCAG-3′) and Tru806R (5′-CGCTCTTCCGATCTGACGGACTACHVGGGTWTCTAAT-3′) were used as the primer pair. Inhibitory primers were designed using Primer3Plus (https://www.bioinformatics.nl/cgi-bin/primer3plus/primer3plus.cgi)^[10]^, and the specificity was checked by Primer-BLAST (https://www.ncbi.nlm.nih.gov/tools/primer-blast/) with default settings^[11]^. Sequences of the inhibitory primers are 5′-GTCCGGTGTGAAAGTCCATC-3′ (for the family *Bifidobacteriaceae*), 5′-TGKSWGTCTTGAGTACAGTAGAGG-3′ (for the genus *Bacteroides*), 5′-CGAAGCCCCCGGAAC-3′ (for *Collinsella aerofaciens*), and 5′-GCGGGAAGACAAGTTGGAA-3′ (for *Faecalibacterium prausnitzii*). The primers with 5′-biotinylation were synthesized by Eurofins Genomics Inc. (Tokyo, Japan), and dissolved in TE buffer [10 mM Tris-HCl (pH 8.0) and 1 mM EDTA]. The composition of the PCR mixture is shown in Table 1. The amplification was carried out by preheating at 95 °C for 2 min, followed by 35 cycles of 98 °C for 10 s, 55 °C for 10 s, and 72 °C for 10 s. The PCRs were performed in three separate tubes for each sample.

**Table 1:** Composition of the first PCR mixture

**Reagent**	**Volume (μL)**	
	**Control**	**DRIP**
Fecal DNA (9-14 ng/μL)	2	2
PrimeSTAR max premix (2×)	10	10
Tru357F (10 μM)	0.4	0.4
Tru806R (10 μM)	0.4	0.4
Biotinylated inhibitory primer (100 μM)	0	0.4 (per primer)
Water	7.2	Up to 20

DRIP: Deeper Resolution using an Inhibitory Primer.

#### Removal of the biotinylated short amplicon

The DNA fragments amplified during the first PCR were purified using the Wizard SV Gel and PCR Clean-Up System (Promega, Madison, WI, USA). The elution was carried out using 60 μL of water. The streptavidin beads (Magnosphere MS300/Streptavidin, JSR Life Sciences, Tokyo, Japan) suspended in 50 μL of 2 × binding buffer [1 × conc. = 10 mM Tris-HCl (pH 7.4), 0.5 mM EDTA, 1 M NaCl, and 0.05% Tween-20] were added to the eluate (50 μL). The mixtures were kept at room temperature for 10 min on an inverting rotator. Subsequently, the tubes were spun down and put on a magnetic stand for 1 min. The resulting supernatant was recovered and purified using the Wizard SV Gel and PCR Clean-Up System. Removal of the biotinylated, short amplicon was checked using the QIAxcel system (Qiagen, Valencia, CA, USA) [Supplementary Figure 1]. The triplicate PCR products for each sample were then combined into one tube.

#### The second PCR

One microliter of the combined eluate pool was used for the second PCR using barcode primers adapted for Illumina Miseq (forward primer: 5′-AATGATACGGCGACCACCGAGATCTACACXXXXXXXXACACTCTTTCCCTACACGACGCTCTTCCGATCTCTG-3′, reverse primer: 5′-CAAGCAGAAGACGGCATACGAGATXXXXXXXXGTGACTGGAGTTCAGACGTGTGCTCTTCCGATCTGAC-3′, where X represents a barcode base). Amplification was carried out with the TaKaRa Ex Taq HS Kit (TaKaRa Bio) as described previously^[8]^. The reaction conditions were as follows: preheating at 94 °C for 3 min, followed by 8 cycles of 94 °C for 30 s, 50 °C for 30 s, and 72 °C for 30 s, with a terminal extension at 72 °C for 5 min. All sequencing libraries were quantified using the KAPA Library Quantification Kit for Illumina Platforms (Roche, Basel, Switzerland).

#### Sequencing and taxonomic classification

Equimolar amounts of the libraries were used for sequencing with Miseq reagent kit v2 (300 cycles) on the Miseq platform (Illumina Inc., Sandiego, CA, USA). Following the acquisition of Illumina paired-end reads, the bowtie-2 program (ver. 2.2.6)^[12] ^was used to remove the reads mapped to PhiX 174 sequence and Genome Reference Consortium human build 37 (GRCh37). Unpaired reads were then removed with a custom Perl script. The 16S rRNA gene sequences were analyzed using the QIIME2 software package version 2017.10 (http://qiime2.org). Potential chimeric reads were removed using DADA2^[13]^, and then 30 and 90 bases of the 3′ region of the forward and reverse reads were trimmed, respectively. Taxonomic classification was performed in QIIME2 software using a Naïve Bayes classifier trained on Greengenes 13.8, with a 99% threshold for operational taxonomic units based on full-length sequences.

### Quantitative PCR

Quantitative PCR (qPCR) was carried out using TB Green Premix Ex Taq^TM^ II kit (Takara Bio) with CronoSTAR 96 Real-Time PCR System (Clontech Laboratories, Mountain View, CA, USA). The primer pairs used were: 5′-ACTCCTACGGGAGGCAGCAGT-3′ and 5′-TATTACCGCGGCTGCTGGC-3′ for total 16S rRNA gene amplification^[14]^, and 5′-AGTGACGGCTAACTACGTGC-3′ and 5′-TTCCAACTTGTYYTCCCGCC-3′ for *F. prausnitzii*-specific amplification. The latter primer pair was designed by Primer-Blast based on the sequences of two ASVs classified as *F. prausnitzii* in our 16S metagenome analysis. The specific amplification of *F. prausnitzii* was confirmed by direct sequencing of the amplicon derived from sample J. The melting curves obtained for other samples were the same as that for sample J. The reaction consisted of denaturation at 95 ºC for 30 s, followed by 45 cycles of 95 ºC for 5 s and 64 ºC for 30 s. The gene copy numbers were calculated based on the standard curves created using a serial dilution of genomic DNA of *F. prausnitzii*. The means of technical duplicate are shown. If no amplification was observed, the lowest value of the standard curve was adopted.

### Statistical analyses

Statistical analyses were performed using R 4.1.2^[15]^. The Hmisc^[16]^ and vegan^[17]^ packages were used to calculate Bray-Curtis dissimilarity and perform Pearson’s correlation coefficient analysis.

## RESULTS

### Conceptualization and experimental proof of the 16S metagenome-DRIP method

**Figure 1:**
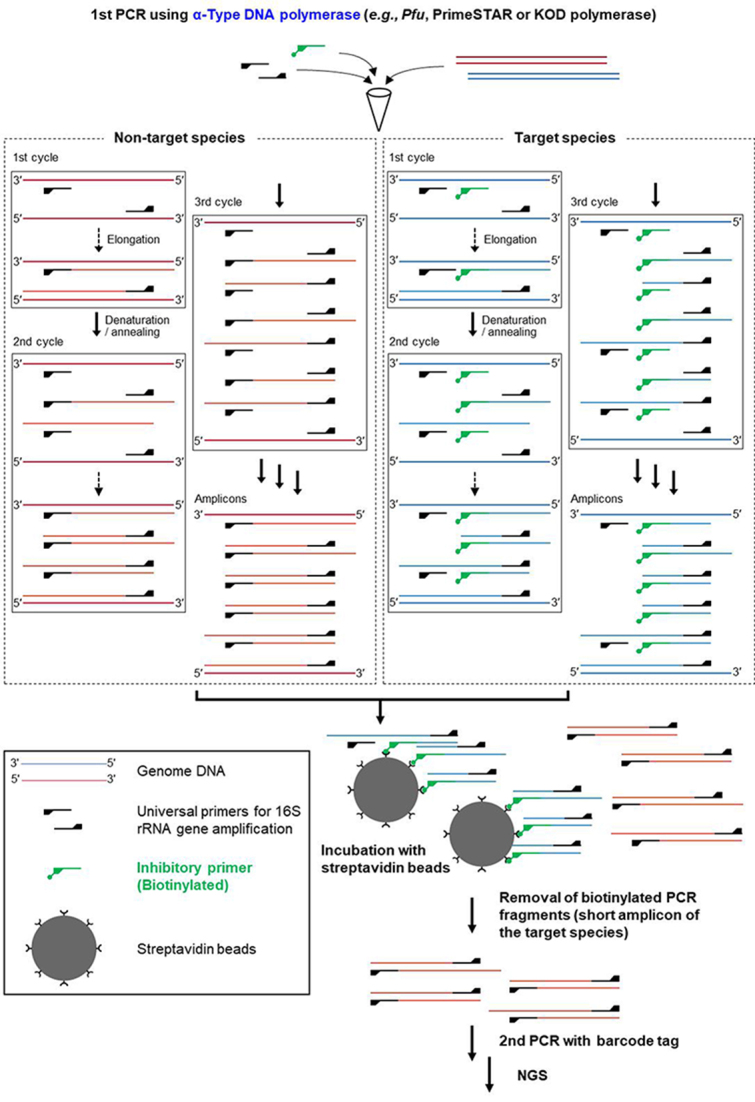
Schematic representation of the 16S metagenome-DRIP analysis. Amplification of the 16S rRNA genes of non-target (left) and target (right) species in the presence of an inhibitory primer specific for the target species. The biotinylated inhibitory primer (green) anneals to an internal site of the amplification target region, which leads to an aberrant short amplicon formation of the 16S rRNA gene of the target species. The short amplicon with the biotin-tag is bound by streptavidin beads and thereby removed from the first PCR mixture. The subsequent procedure is identical to the conventional 16S metagenome analysis. NGS: Next-generation sequencing.

Figure 1 shows the concept and scheme of the 16S metagenome-DRIP analysis. The DRIP method involves two additional experimental procedures to the scheme for the conventional 16S metagenomic analysis during the first PCR so that the PCR amplicon derived from a target major species can be removed from the reaction mixture. The first is the addition of a biotinylated-inhibitory primer, which is complementary to a sequence in the variable region of the 16S rRNA gene of a target species. The addition of the inhibitory primer results in an aberrant short-length DNA fragment amplification of the target species [Figure 1, right panel]. The second is the removal of the inhibitory primer-derived biotinylated short amplicon by using streptavidin beads. The tight binding between biotin and streptavidin is frequently utilized in biochemical experiments to capture or remove labeled products. The detailed reaction conditions are described in the Methods Section. The subsequent procedures, i.e., the second PCR and sequencing, are completely the same as conventional 16S metagenome techniques. The DRIP method is thus quite simple. However, it requires the use of α-type DNA polymerases such as *Pfu*, PrimeSTAR, and KOD for amplification. When Pol-I type polymerases such as *Taq* DNA polymerase are used, the inhibitory primer is degraded by the 5′→3′ exonuclease activity of the enzymes, a feature utilized for TaqMan-based technologies. The lack of the 5′→3′ exonuclease activity in α-type DNA polymerases makes the elongation reaction pause (correctly, idle) when the polymerases encounter the biotinylated primer annealed to the target template DNA. Amplification of non-target species would theoretically proceed normally.

**Figure 2:**
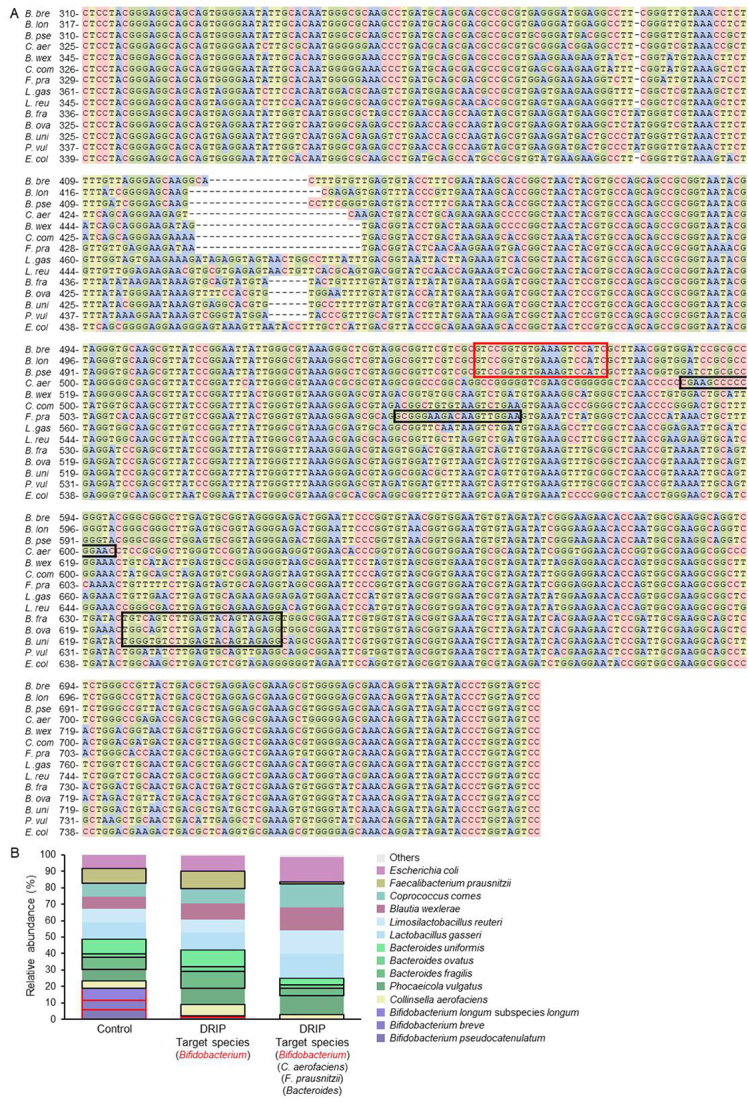
Proof-of-concept experiment using a mock sample. (A) The sequence alignment of the V3-V4 region of the 16S rRNA genes of 14 bacterial species (*B. breve *JCM 1192, *B. longum *subsp.* longum *JCM 1217, *B. pseudocatenulatum* JCM 1200, *C. aerofaciens* JCM 10188, *B. wexlerae* JCM 17041, *C. comes* ATCC 27758, *F. prausnitzii* JCM 31915, *L. gasseri* JCM 1131, *L. reuteri* JCM 1112, *B. fragilis* JCM 11019, *B. ovatus* JCM 5824, *B. uniformis* JCM 5828, *P. vulgatus* JCM 5826, and *E. coli* MG1655). The inhibitory primer sequences, which correspond to the V4 region, are boxed in red and black. (B) A mixture of the 14 bacterial genomes was subjected to the conventional 16S metagenome (Control) and 16S metagenome-DRIP (DRIP) analyses. The relative abundances of 14 species are shown in different colors. DRIP: Deeper Resolution using an Inhibitory Primer.

As a proof-of-concept study, we first conducted a mock experiment using a mixture of equal amounts of purified genomes of 14 bacterial species: four from the phylum Actinomyceota (formerly Actinobacteria) (*Bifidobacterium breve*, *Bifidobacterium longum* subspecies* longum*,* Bifidobacterium pseudocatenulatum*, and* Collinsella aerofaciens*), five from the phylum Bacillota (Firmicutes) (*Blautia wexlerae*,* Coprococcus comes*,* Faecalibacterium prausnitzii*,* Lactobacillus gasseri*, and* Limosilactobacillus reuteri*), four from the phylum Bacteroidota (Bacteroidetes) (*Bacteroides fragilis*, *Bacteroides ovatus*, *Bacteroides uniformis*, and *Phocaeicola vulgatus*), and one from the phylum Pseudomonadota (Proteobacteria) (*Escherichia coli*). These bacteria show relatively high prevalence and/or dominance in the human gut microbiota^[18]^ or are used as probiotics worldwide. Among these bacteria, the genus *Bifidobacterium* was first selected as a target for DRIP as it is a frequently dominant taxon in breastfed infant guts^[19]^, thereby potentially masking microbial diversity. An inhibitory primer specific for the genus *Bifidobacterium* was designed based on the sequence alignment of the V3-V4 region of the 16S rRNA genes of the 14 species. The designed primer corresponds to the V4 region [Figure 2A], and its specificity was evaluated by Primer-BLAST.

With the conventional 16S metagenome analysis (hereafter referred to as Control), the relative abundances of the 14 species were comparable, ranging between 7.1% and 8.7% (mean ± SD: 7.7 ± 0.84%). An exception was *B. ovatus*, whose relative abundance was 2.2%. When the DRIP method was applied (hereafter referred to as DRIP), the abundances of *B. longum* subsp. *longum*,* B. breve*, and* B. pseudocatenulatum* drastically decreased from 7.2%, 5.9%, and 5.9% to 0.5%, 0.5%, and 1.3%, respectively. At the genus level, relative abundance decreased from 19.0% (2618 reads) to 2.3% (363 reads). To compensate for the decrease of *Bifidobacterium* reads, the relative abundances of the other 10 species slightly increased by 1.0-1.6-fold [Figure 2B]. The abundance of *B. ovatus* was again lower compared to other species, which suggested an intrinsic PCR bias. The total read counts obtained were comparable between the Control (13,779) and DRIP (15,747) groups. The microbial communities (with *Bifidobacterium* omitted) did not differ considerably between the two groups (Bray-Curtis dissimilarity: 0.064). Biotinylation of the inhibitory primer and the following removal of a short amplicon derived from the target species (see Supplementary Figure 1) are critical for obtaining a comparable number of reads in the DRIP method, as the carry-over of the short amplicon compromises DNA quantification prior to the second PCR as well as interferes with efficient amplification during the second PCR. The target species-derived short amplicon formed during the first PCR contains a complementary sequence at one end of the DNA fragment to the barcode-tagged primer used in the second PCR [Figure 1, right panel]. Indeed, in a separate experiment in which a sample with the relative abundance of *Bifidobacterium* of 60% was used as the template in the first PCR and the resulting reaction products were directly used for the second PCR without the removal process, the read counts decreased to one-third of the Control sample (4568/12,506), although the relative abundance of *Bifidobacterium* decreased to 1.1%, as expected.

We then examined if multiple primers can be simultaneously used with the DRIP method. Three inhibitory primers were additionally designed to inhibit the amplification of 16S rRNA genes of *C. aerofaciens*, *F. prausnitzii*, and the genus *Bacteroides*, and they were added to the reaction mixture together with the genus* Bifidobacterium*-specific primer (see the "*Methods*" Section and Table 1). In the presence of four inhibitory primers, the total read counts slightly decreased to 10,242. The relative abundances of the *Bifidobacterium* and *Bacteroides *genera decreased from 19.0% and 18.3% to 0.3% and 10.4%, respectively, while the relative abundances of *C. aerofaciens* and *F. prausnitzii* decreased from 4.3% and 9.4% to 2.7% and 1.3%, respectively. The Bray-Curtis dissimilarity was 0.066 between the target species-omitted Control and DRIP communities. The results indicate that the efficacy of the DRIP method is dependent on the specificity of the designed primers. Nonetheless, the presence of multiple inhibitory primers in the PCR mixture did not markedly affect the amplification of non-target species. Taken together, the data strongly suggest that the DRIP method can be applied without substantially disrupting other processes in the 16S metagenomic analysis.

### Application of 16S metagenome-DRIP to infant gut microbiota analysis

**Figure 3:**
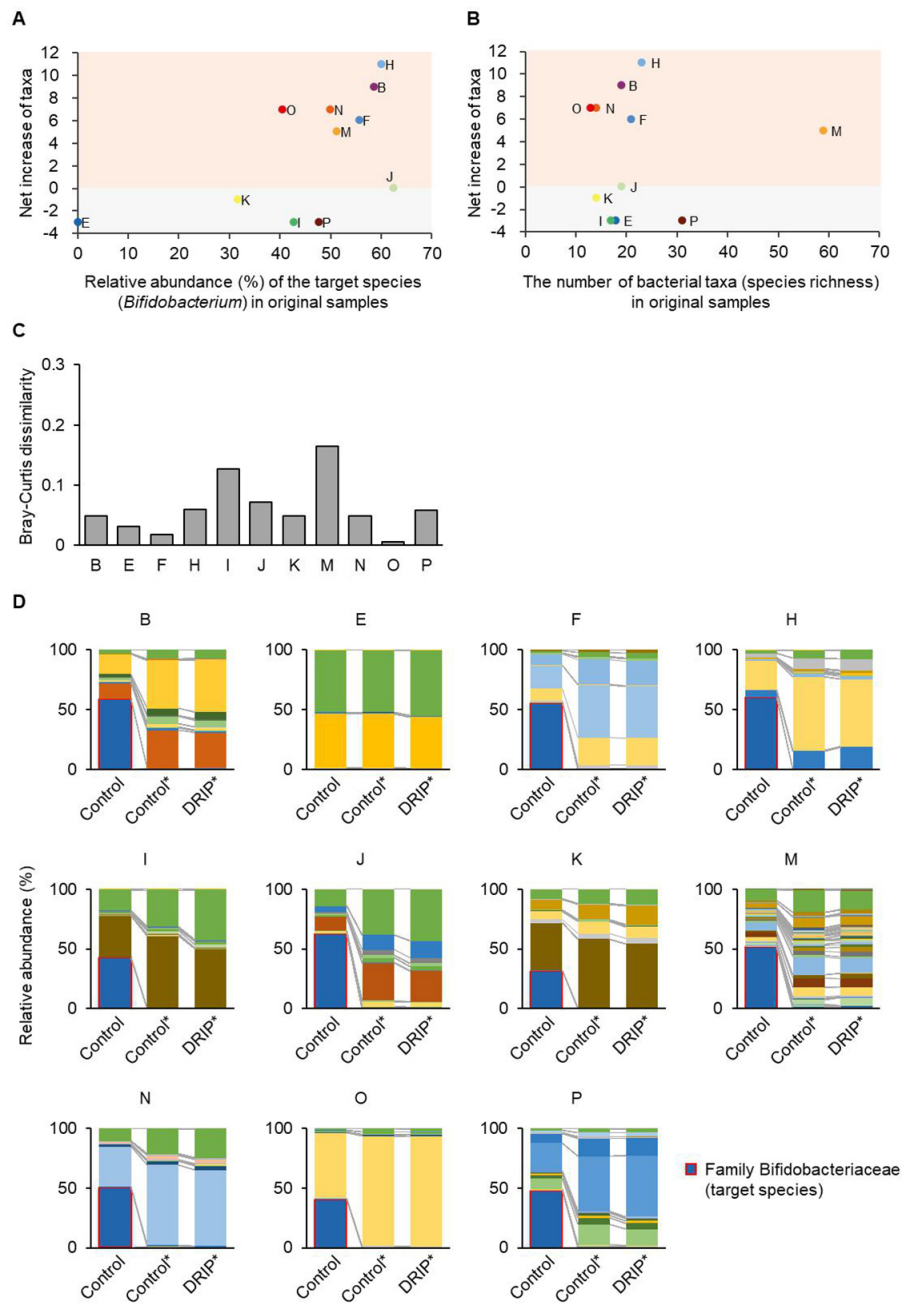
Validation of 16S metagenome-DRIP method using infant fecal samples. (A and B) Net increase of bacterial taxa is correlated with the relative abundance of the target species *Bifidobacterium* (A) but not with the number of bacterial taxa (species richness) (B) in samples (Pearson’s correlation coefficient analysis). Subject IDs are indicated (see Table 2). (C) Bray-Curtis dissimilarity indices between the Control and DRIP groups. The values were calculated by omitting the target species (*Bifidobacterium*). (D) The relative abundances (%) of bacterial taxa detected in infant fecal samples analyzed by the conventional 16S metagenome (Control) and 16S metagenome-DRIP (DRIP) methods. Asterisks (*) indicate that the reads corresponding to the target species (*Bifidobacterium*) were omitted for drawing the bar charts. Different colors are given to different taxa. The target species (*Bifidobacterium*) is shown in dark blue with a red frame. DRIP: Deeper Resolution using an Inhibitory Primer.

The DRIP method was applied to the fecal DNA of 11 infants, and the results were compared between the Control and DRIP groups. The average total read counts were comparable between the two groups (36,073 *vs.* 34,778 per sample) (Paired *t*-test, *P* = 0.85), while the average read counts corresponding to family *Bifidobacteriaceae* decreased from 16,657 to 1718 (Paired *t*-test, *P* = 0.014) [Table 2]. It should also be mentioned that the read counts were comparable between the two groups during the filtering process, indicating that background noise amplification did not occur in the first PCR of the DRIP method [Supplementary Table 1]. In the following section, we focus on non-target species, i.e., the ASVs assigned to the family *Bifidobacteriaceae *were excluded from the analysis unless otherwise indicated. As summarized in Table 2, the number of bacterial taxa detected in the DRIP group was increased in six infant samples, decreased in four samples, and unchanged in one sample, as compared to that in the Control group. When examining individual samples, the number of newly appeared taxa in DRIP was 7 (4 and 8.5) (median, first and third quartiles), while the number of taxa that disappeared in DRIP was 2 (1.5 and 5.5). Accordingly, the net increase in bacterial taxa was 5 (-2 and 6.5) by applying the DRIP method. Notably, the net increase in bacterial taxa seemed to be positively correlated, although statistically not significant, with the relative abundance of the target species in the Control sample, i.e., the intrinsic abundance of* Bifidobacterium* in samples (*r* = 0.554, *P* = 0.077, Pearson’s correlation coefficient analysis) [Figure 3A], while it showed no correlation with the number of bacterial taxa detected in the Control group, i.e., the intrinsic species richness in samples (*r* = 0.048, *P* = 0.89) [Figure 3B]. These results indicate that the increased detection rate of new taxa is attributable to the DRIP method and not caused by PCR bias during amplification. The Bray-Curtis dissimilarity indices were 0.049 (0.032 and 0.072) between the Control and DRIP groups [Figure 3C]. Consistent with this, the relative abundances (%) of species other than the target taxon (*Bifidobacterium*) were similar between the two groups among 11 infant samples [Figure 3D]. These results strongly suggest that the DRIP method essentially affects the target species detection and does not compromise the intrinsic microbiota composition and PCR amplification, given that the primer specificity is high for the target taxon.

**Table 2:** Summary of the 16S metagenome analysis of 11 infant feces with and without DRIP^a^

**ID**	**Detected species (Control)**	**Detected species (DRIP)**	**Newly appeared species by DRIP**	**Disappeared species by DRIP**	**Net increase by DRIP**	**Read counts (Control)**	**Read counts (DRIP)**
B	19	28	11	2	9	14,794	17,570
E	18	15	4	7	-3	26,002	24,040
F	21	27	8	2	6	84,827	99,917
H	23	34	12	1	11	21,032	31,590
I	17	14	2	5	-3	12,112	15,583
J	19	19	6	6	0	48,183	54,536
K	14	13	0	1	-1	20,422	18,580
M	59	64	18	13	5	12,249	11,108
N	14	21	7	0	7	13,297	19,881
O	13	20	9	2	7	122,692	70,204
P	31	28	4	7	-3	21,200	22,278

^a^The analysis does not include ASVs assigned to the family *Bifidobacteriaceae*. DRIP: Deeper Resolution using an Inhibitory Primer.

### Analysis of ASVs unique to the Control and DRIP groups

In total, 298 and 391 ASVs were detected in the Control and DRIP groups, respectively, in our analysis [Supplementary Table 2]. Among them, 16 ASVs of the Control group and 18 ASVs of the DRIP group were assigned to the family *Bifidobacteriaceae*. In total, 115 ASVs (including seven Bifidobacteriaceae) were unique to the Control group, while 208 ASVs (including nine Bifidobacteriaceae) were unique to the DRIP group. The remaining 183 ASVs (including nine Bifidobacteriaceae) were shared between the two groups. The ASVs unique to each of the two groups were then manually analyzed with the BLASTN program using rRNA/ITS database of NCBI (https://blast.ncbi.nlm.nih.gov/Blast.cgi) [Supplementary Tables 3 and 4]. When the sequence coverage and/or the sequence identity was lower than 97% in the retrieved results, the corresponding sequences were re-analyzed against the RefSeq (nr/nt) database. As a result, 12 ASVs of the Control group and 10 ASVs of the DRIP group were found to be potential chimeric 16S rRNA gene sequences that were not removed by DADA2^[13]^, while 3 ASVs of the Control group and 1 ASV of the DRIP group were assigned to be protein sequences. Interestingly, 25 and 76 ASVs of the Control and DRIP groups, respectively, showed ≥ 97% identity to uncultured bacterial 16S rRNA genes with coverage of ≥ 97%. These results indicate the effectiveness of the DRIP method in terms of increasing the microbiota resolution without compromising other analytical processes.

### Accuracy of 16S metagenome-DRIP for relative abundance estimation for a minority species

**Figure 4:**
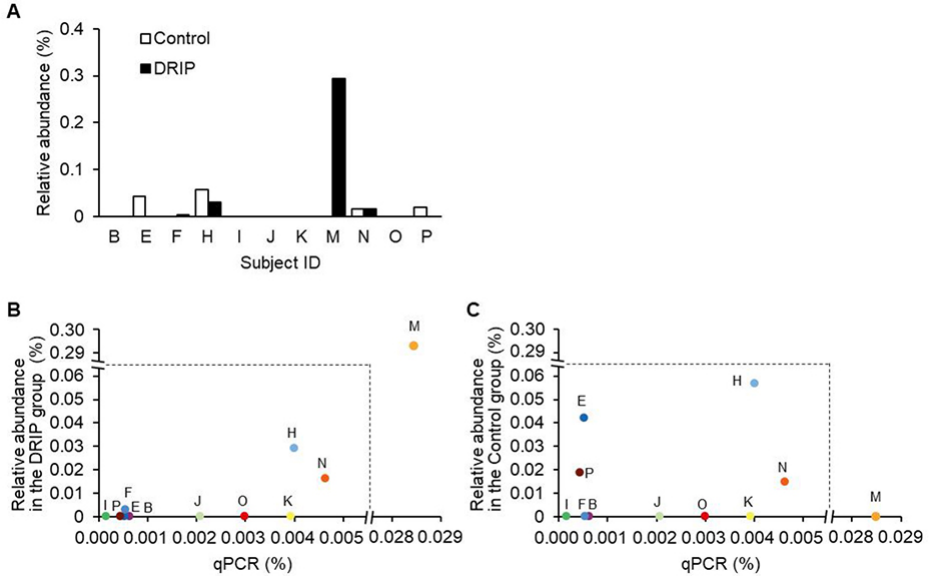
Accuracy of 16S metagenome-DRIP for estimating the abundance of a minority species. (A) The relative abundances (%) of *F. prausnitzii* estimated from the results of the conventional 16S metagenome (white bars) and 16S metagenome-DRIP (black bars) analyses. (B and C) The relative abundances (%) of *F. prausnitzii* determined by qPCR were compared with those deduced from the data of 16S metagenome-DRIP (B) or the conventional 16S-metagenome (C) analysis. Pearson’s correlation coefficient analysis was used to evaluate the data. Subject IDs are indicated (see Table 2). DRIP: Deeper Resolution using an Inhibitory Primer; qPCR: quantitative PCR.

Gonzalez *et al.* reported the risk of under- or overestimating the relative abundance of minority species in 16S metagenome analysis due to unignorable bias during the PCR amplification process for low abundance DNA^[20]^. We, therefore, examined the accuracy of the DRIP method for estimating the relative abundance of minority species. For analysis, we selected *F. prausnitzii* as it was the newly appeared, most abundant species in sample M after applying the DRIP method. Sample M showed the highest dissimilarity in the Bray-Curtis analysis [Figure 3C]. The relative abundances of *F. prausnitzii* calculated based on the results of 16S metagenome analysis [Figure 4A] were compared with the results obtained by qPCR. The results reveal that the relative abundance calculated from DRIP is positively correlated with the results of qPCR with strong statistical significance (*r* = 0.987, *P* = 1.8 × 10^-8^, Pearson’s correlation coefficient) [Figure 4B]. In contrast, no significant correlation was detected between qPCR results and the conventional 16S metagenome data (Control) (*r *= -0.158, *P* = 0.64) [Figure 4C]. The results strongly suggest that the DRIP method can detect the abundances of minority species more accurately than the conventional 16S metagenome analysis. A more accurate estimation of the entire microbial community may be possible by replacing the abundances of non-target species obtained through conventional 16S metagenomic analysis with the abundances obtained through the DRIP method, after normalizing the community composition using the read counts between the two methods.

## DISCUSSION

In this paper, we describe the conceptualization, a proof-of-concept experiment using a mock sample, and an application to infant samples of the 16S metagenome-DRIP method. Our results demonstrate the utility of the DRIP method for efficient and accurate minority species detection when combined with the conventional 16S metagenome analysis. However, there are several caveats to be mentioned.

*Selection of DNA polymerase*: Pol-I type DNA polymerases, e.g., r*Taq*, are often used during the first PCR in NGS. However, 16S metagenome-DRIP requires the use of α-type enzymes such as *Pfu*, PrimeSTAR, and KOD. The second PCR can be conducted using the same methods as conventional 16S metagenome analyses.

*Specificity*: Efficient inhibition of DNA amplification of a target species depends on primer specificity. The inhibitory primer designed for the genus *Bifidobacterium* worked well for both the mock sample [Figure 2B] and infant fecal samples [Figure 3], and the primer designed for *F. prausnitzii* also inhibited normal amplification for the species in the mock sample [Figure 2B]; however, the inhibition efficacy of the two other primers designed for the genus *Bacteroides* and *C. aerofaciens* was considerably lower [Figure 2B]. Primer design could also be sample-dependent. If the community comprises only taxonomically close bacterial species, it would be quite difficult to design a specific primer to inhibit the target species. We did not address how many mismatches of base pairs are required or permitted for primer design, which should govern the effectiveness of this method.

*Target species*: 16S metagenome-DRIP would be effective only when the microbiotas to be examined contain at least one dominant species in the community. Indeed, the net increase in the number of bacterial taxa detected by applying 16S metagenome-DRIP was positively correlated with the relative abundance (%) of the genus *Bifidobacterium* in the infant samples [Figure 3A]. Additionally, the DRIP method is not applicable when no prior information is available for community compositions in the samples to be analyzed. In such cases, pre-analysis of microbiotas using the conventional 16S metagenome is required for designing appropriate inhibitory primers.

*Stochastic effect*: Although the total minority species detection rate (detection rate of different ASVs) increased in DRIP, the appearance of new species (and ASVs) seems to be subject to stochastic effects. In fact, a net decrease in the number of the detected species was observed in several samples.

We hope that the DRIP method will enhance minority species detection in environmental samples and help us understand how minority species influence microbiota formation in natural environments.

## DECLARATIONS

### Availability of data and materials

The amplicon sequences of the V3-V4 region of the 16S rRNA gene with and without DRIP have been deposited in the DDBJ databank under the accession number DRA013692. All materials except for infant feces are available upon request.

### Copyright

© The Author(s) 2022.
