# Supplementary material for: A simple method that enhances minority species detection in the microbiota: 16S metagenome-DRIP (Deeper Resolution using an Inhibitory Primer)
Source: Microbiome Res Rep. 2022 May 30;1(3):20. doi: 10.20517/mrr.2022.08 (PMC10688780; doi:10.20517/mrr.2022.08)
Supplement: Supplementary file 1 [file mrr-1-3-20-SupplementaryMaterials.zip › 4916-SupplementaryMaterials/4916-Supplementary Material (Figure 1).pdf]

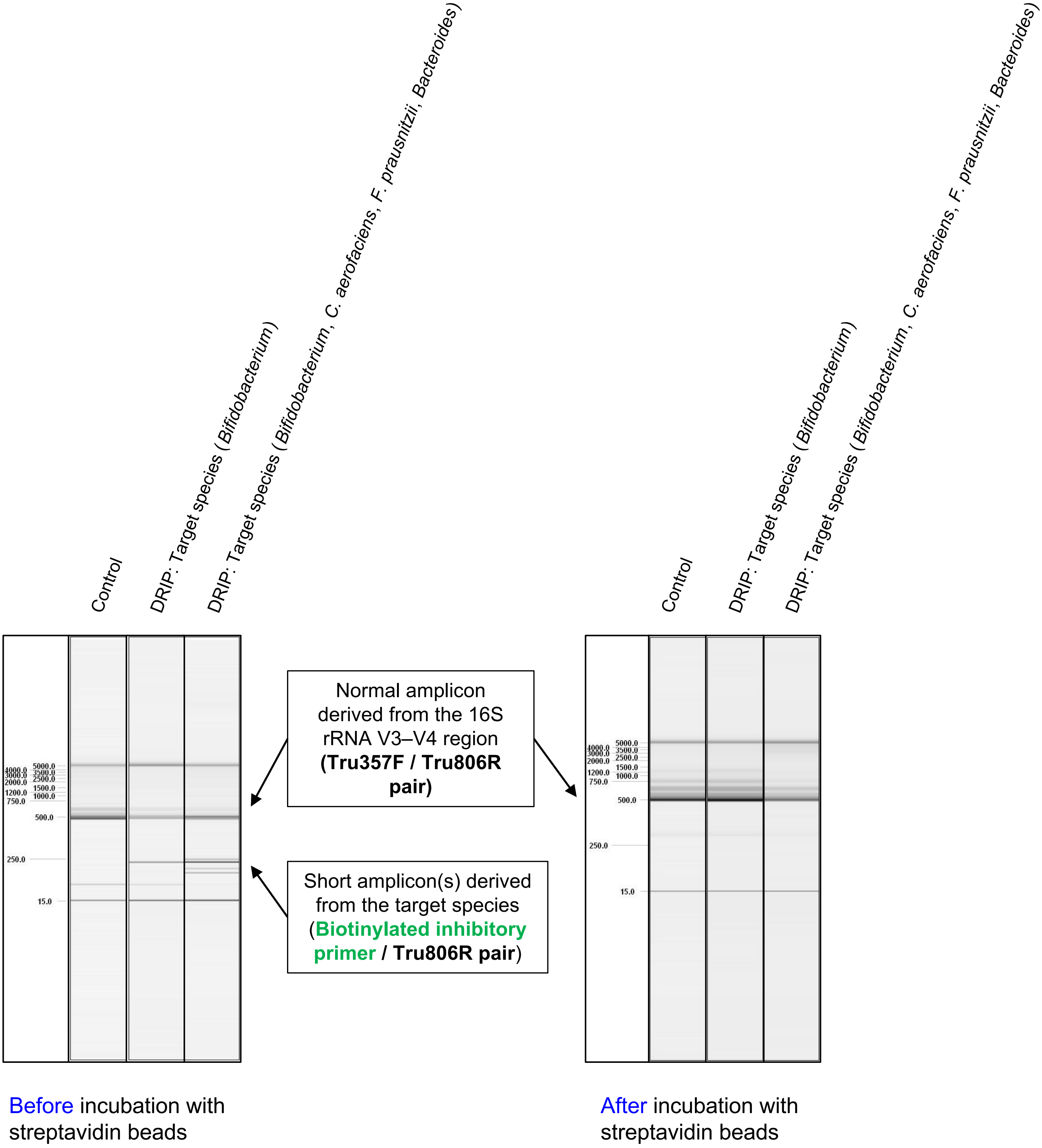

Supplementary Figure 1. Removal of the short amplicon(s) derived from the target species using streptavidin beads. The results of capillary electrophoreses of PCR products obtained in the 1st PCR step, before (left panel) and after (right panel) incubation with streptavidin beads, are shown. Left lanes: the conventional 16S-metagenome PCR (Control); middle lanes: 16S metagenome-DRIP PCR with the inhibitory primer for the genus *Bifidobacterium*; right lanes, 16S metagenome-DRIP PCR with four inhibitory primers for the *Bifidobacterium* and *Bacteroides* genera, *C. aerofaciens*, and *F. prausnitzii*. Streptavidin beads were used to capture the biotin-labeled short amplicon.
